# 

**Published:** 1899-08

**Authors:** 


### Contributors to Volume XXXIX.

**New Series—August, 1899-July, 1900.**

---

| | |
|---|---|
| ANGELL, EDWARD B. | Rochester. |
| BEHAN, A. L. | Canandaigua. |
| BISSELL, WILLIAM G. | Buffalo. |
| CLAYTON, MARY | Buffalo. |
| CLEMESHA, J. C. | Buffalo. |
| CLINTON, MARSHALL | Buffalo. |
| COAKLEY, JOHN B. | Buffalo. |
| COLTON, ALBERT J. | Buffalo. |
| CONKLIN, W. L. | Rochester. |
| COTT, GEORGE F. | Buffalo. |
| COULTER, C. W. | Oil City. |
| DAGGETT, BYRON H | Buffalo. |
| DENNIS, FREDERIC H. | New York. |
| DOWD, J. HENRY | Buffalo. |
| DWYER, THOMAS F | Buffalo. |
| ELLIS, CHARLES E | Sherman. |
| ELSNER, S. L. | Rochester. |
| FINERTY, JOHN J. | Buffalo. |
| FRONCZAK, FRANCIS E. | Buffalo. |
| FRYE, MAUD J. | Buffalo. |
| GRAM, FRANKLIN C | Buffalo. |
| GREENE, GEORGE WALTON | Auburn. |
| GROVE, BENJAMIN H. | Buffalo. |
| HALL, A. L | Fair Haven. |
| HAYD, HERMAN E | Buffalo. |
| HEATH, WILLIAM H. | Buffalo. |
| HIMMELSBACH, GEORGE A | Buffalo. |
| HINKEL, FRANK WHITEHILL | Buffalo. |
| HOPKINS, HENRY R | Buffalo. |
| HOWE, LUCIEN | Buffalo. |
| HOWELL, STEPHEN Y. | Buffalo. |
| HUBBELL, ALVIN A | Buffalo. |
| ILL, EDWARD J | Newark. |
| INGERSOLL, J. M. | Rochester. |
| JACK, GEORGE N. | Depew. |
| KING, CLARENCE | Machias. |
| KRAUSS, WILLIAM C. | Buffalo. |
| LE BRETON, PRESCOTT | Buffalo. |
| LONG, ELI H | Buffalo. |

### CONTRIBUTORS TO VOLUME XXXIX.

| | |
|---|---|
| LOVELAND, B. C | Syracuse. |
| LYTLE, ALBERT T | Buffalo. |
| McGUIRE, FRANK W. | Buffalo. |
| MILLENER, FREDERICK H. | Buffalo. |
| MILLER, JOHN A. | Buffalo. |
| MOONEY, E. L | Syracuse. |
| PARK, ROSWELL | Buffalo. |
| PARMENTER, JOHN | Buffalo. |
| PETERSON, FREDERICK | New York. |
| POTTER, WILLIAM WARREN | Buffalo. |
| PUTNAM, JAMES WRIGHT | Buffalo. |
| RANNEY, AMBROSE L | New York. |
| REED, CHARLES A. L | Cincinnati. |
| RICKETTS, EDWIN | Cincinnati. |
| SATTERLEE, RICHARD H | Buffalo. |
| SPRATLING, WILLIAM P. | Sonyea. |
| SNOW, SARGENT F | Syracuse. |
| SOBLE, NATHAN W | Rochester. |
| STOCKTON, CHARLES G. | Buffalo. |
| SUTTON, R. STANSBURY | Pittsburg. |
| TAYLOR, W. G. | Buffalo. |
| THORNDIKE, PAUL | Boston. |
| WENDE, ERNEST | Buffalo. |
| WENDE, GROVER W | Buffalo. |
| WILSON, NELSON W. | Buffalo. |
| YOUNG, A. A. | Newark. |

AMERICAN ASSOCIATION OF OBSTETRICIANS AND GYNECOLOGISTS.
BUFFALO ACADEMY OF MEDICINE.
MEDICAL ASSOCIATION OF CENTRAL NEW YORK.
MEDICAL SOCIETY OF THE COUNTY OF CHAUTAUQUA.
MEDICAL SOCIETY OF THE COUNTY OF ERIE.

### BOOKS RECEIVED.

The Mineral Waters of the United States and Their Therapeutic Uses. With an account of the various mineral spring localities, means of access, etc. By James K. Crook, A. M., M. D., Adjunct Professor of Clinical Medicine and Physical Diagnosis at the New York Post-Graduate Medical School, etc. In one 8vo volume of 580 pages. Cloth, $3.50, net. Philadelphia and New York: Lea Brothers & Co. 1899.

A Manual of Diseases of the Nervous System. By Sir W. R. Gowers, M. D., F. R. C. P., F. R. S., Consulting Physician to University College Hospital; Physician to the National Hospital for the Paralysed and Epileptic, Queen Square. Third edition, revised and enlarged. Edited by Sir W. R. Gowers and James Taylor, M. A., M. D., F. R. C. P., Senior Assistant Physician to the National Hospital for the Paralysed and Epileptic, Queen Square; Physician to the North-eastern Hospital for Children and to the National Orthopedic Hospital. Vol. I., Diseases of the Nerves and Spinal Cord. With 192 illustrations. Octavo. Price, $4.00, net. Philadelphia: P. Blakiston's Son & Co., 1012 Walnut Street. 1899.

The Mechanics of Surgery. Comprising detailed descriptions, illustrations and lists of the instruments, appliances and furniture necessary in modern surgical art. By Charles Truax. Octavo, pp. 1,024. Chicago: 1899.

Transactions of the Southern Surgical and Gynecological Association. Eleventh Annual Session, held at Memphis, Tenn., December 6, 7 and 8, 1898. William E. B. Davis, M. D., Secretary.

The Medical Directory of the City of New York. Published by the Medical Society of the County of New York. Price $1.50. New York: Wynkoop, Hallenbeck, Crawford Co., Printers. 1899.

Massage and the Original Swedish Movements. Their application to various diseases of the body. Lectures before the training schools for nurses connected with the Hospital of the University of Pennsylvania, German Hospital, Woman's Hospital, etc. By Kurre W. Ostrom, from the Royal University of Upsala, Sweden. Fourth edition, revised and enlarged, with 105 illustrations. Price, $1.00. Philadelphia: P. Blakiston's Son & Co. 1899.

The Newer Remedies. A Reference Manual for Physicians, Pharmacists and Students. By Virgil Coblentz, A. M., Phar. M., Professor of Chemistry and Physics in the New York College of Pharmacy. Third edition, revised and enlarged; 150 pages. Price, $1.00, net. Philadelphia: P. Blakiston's Son & Co., 1012 Walnut Street. 1899.

